# Journey to the Center of the Fetal Brain: Environmental Exposures and Autophagy

**DOI:** 10.3389/fncel.2018.00118

**Published:** 2018-05-03

**Authors:** Jun Lei, Pilar Calvo, Richard Vigh, Irina Burd

**Affiliations:** Department of Gynecology and Obstetrics, Integrated Research Center for Fetal Medicine, Johns Hopkins University School of Medicine, Baltimore, MD, United States

**Keywords:** fetal brain development, adverse perinatal neurologic outcomes, pregnancy environmental exposures, alcohol, autophagy biomarkers

## Abstract

Fetal brain development is known to be affected by adverse environmental exposures during pregnancy, including infection, inflammation, hypoxia, alcohol, starvation, and toxins. These exposures are thought to alter autophagy activity in the fetal brain, leading to adverse perinatal outcomes, such as cognitive and sensorimotor deficits. This review introduces the physiologic autophagy pathways in the fetal brain. Next, methods to detect and monitor fetal brain autophagy activity are outlined. An additional discussion explores possible mechanisms by which environmental exposures during pregnancy alter fetal brain autophagy activity. In the final section, a correlation of fetal autophagy activity with the observed postnatal phenotype is attempted. Our main purpose is to provide the current understanding or a lack thereof mechanisms on autophagy, underlying the fetal brain injury exposed to environmental insults.

## Physiology

Autophagy is the cellular “self-eating” process by which damaged intracellular proteins, organelles and pathogens are degraded (Carloni et al., 2008; Kadandale and Kiger, 2010). Under physiologic conditions, it provides a degrade and recycle mechanism that releases amino acids, free fatty acids, and monosaccharides for reuse (Zhang F. et al., 2016). Autophagy is a highly conserved pathway common among disparate cladistic classes such as yeast, roundworms, and humans (Levine and Klionsky, 2004). Depending on the pathology, autophagy can offer a beneficial cell salvage pathway. Conversely, it can also act with apoptosis to promote cell death (Bildirici et al., 2012), especially when autophagy activity is extremely elevated (Guha et al., 2016). Autophagy may also inhibit apoptosis by way of mitochondria sequestration (Rocha-Ferreira and Hristova, 2016).

In mammals, there are three types of autophagy: macroautophagy, microautophagy, and chaperone mediated autophagy (Tekirdag and Cuervo, 2018). Macroautophagy involves the synthesis of multilayered vesicles called autophagosomes, which surround intracellular organelles such as mitochondria as well as proteins. The loaded autophagosome fuses with a lysosome, releasing lysosomal proteolytic enzymes that digest the contents of the vesicle (Hamasaki et al., 2013). Microautophagy is a similar process that does not use autophagosome vacuolates. Instead, microautophagy relies on the lysosome invaginating itself to surround and then digest the degradation target (Marzella et al., 1981; Hamasaki et al., 2013). In contrast, the targets of chaperone-mediated autophagy are not surrounded by vesicular structures. This latter process relies on chaperone proteins selecting and marking intracellular proteins which are then translocated across the lysosomal membrane and degraded (Kaur and Debnath, 2015). Of the three types, the most commonly studied and the one forming the basis of this review is macroautophagy, hereon referred to as autophagy.

**Figure 1:**
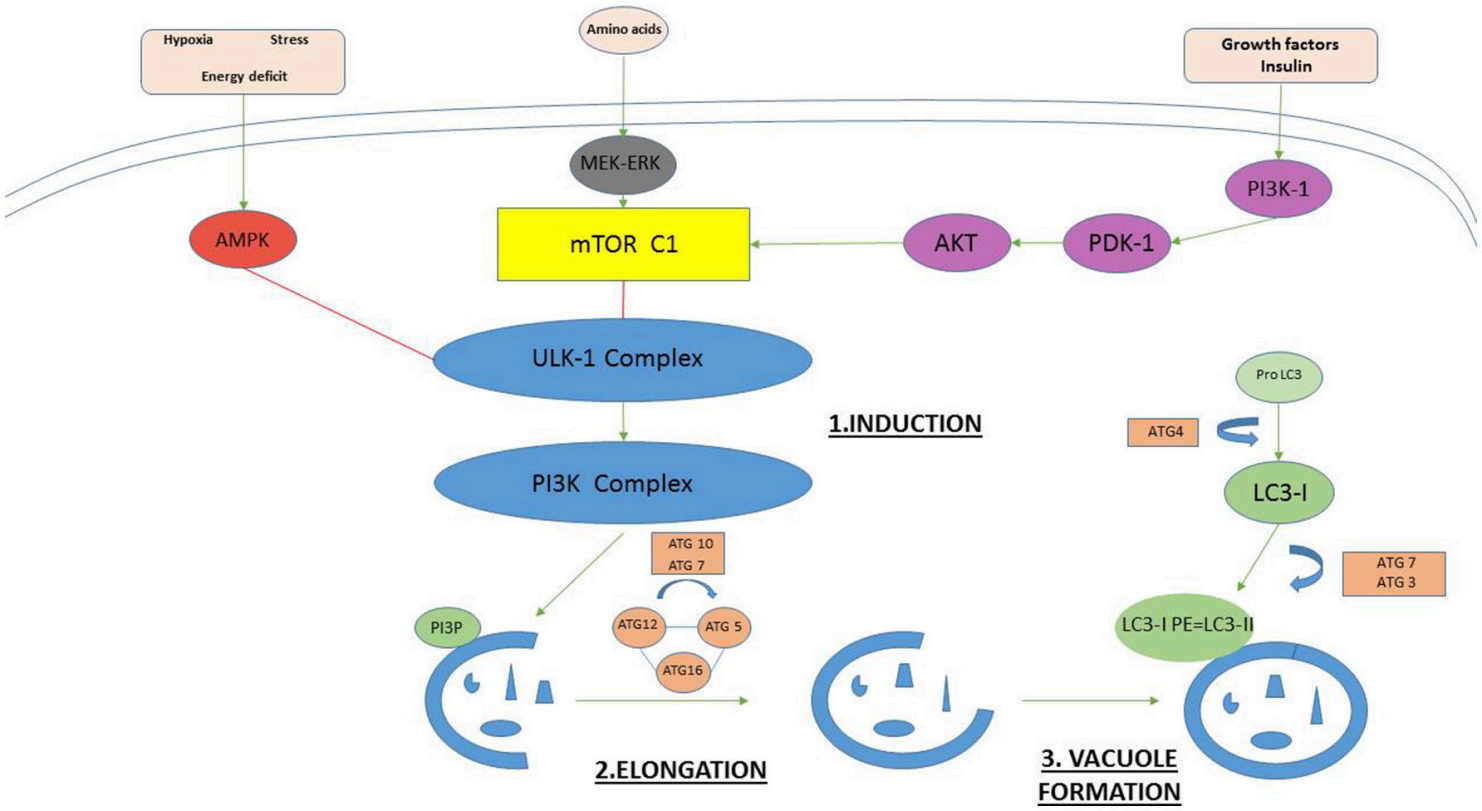
The key proteins in mammalian autophagosome formation. Autophagy can be initiated in two primary ways. The first is via activation of AMPK (adenosine monophosphate activated protein kinase) under hypoxia, stress, and energy deficit. The second is via inhibition of the nutrient sensing system mammalian target of rapamycin (mTOR). Both mechanisms lead to phosphorylation and activation of the Unc-51-like kinase (ULK1) complex that starts inducting formation of the multilamellear phagophore. The ULK1 complex activates the phosphatidylinositol-3 kinase class III (PI3K CIII) complex composed of beclin-1, Atg14, vacuolar protein sorting (VPS) proteins Vps34 and Vps15, which in turn generates phosphatidylinositol 3-phosphate (PI3P) to facilitate membrane elongation. Various Atg proteins join together to form the Atg5-Atg12-Atg16 complex. This complex triggers the cleavage of pro-microtubule-associated protein 1 light chain 3 (LC3) to form LC3-I which is then conjugated to phosphatidylethanolamine (PE) to form LC3-II. LC3-II promotes closure of the vesicle membrane, which is the event that signals the final step in autophagosome vacuole formation.

Autophagy is a strictly controlled process mediated by many proteins (Mizushima et al., 2010; Meschini et al., 2011; Yoshii and Mizushima, 2017). An overview of the main functional protein complexes and their interactions is provided in Figure 1. Autophagy can be initiated in two primary ways. The first is via activation of AMPK (adenosine monophosphate activated protein kinase). The second is via inhibition of the nutrient sensing system mammalian target of rapamycin (mTOR) (Roos et al., 2009). Both mechanisms lead to phosphorylation and activation of the Unc-51-like kinase (ULK1) complex that starts inducting formation of the multilamellear phagophore. The ULK1 complex activates the phosphatidylinositol-3 kinase class III (PI3K CIII) complex composed of beclin-1, autophagy-related protein (ATG) 14, vacuolar protein sorting (VPS) proteins Vps34 and Vps15, which in turn generates phosphatidylinositol 3-phosphate (PI3P) to facilitate membrane elongation. Various Atg proteins join together to form the Atg5-Atg12-Atg16 complex. This complex triggers the cleavage of pro-microtubule-associated protein 1 light chain 3 (LC3) to form LC3-I which is then conjugated to phosphatidylethanolamine (PE) to form LC3-II. LC3-II promotes closure of the vesicle membrane, which is the event that signals the final step in autophagosome vacuole formation.

The importance of autophagy is evident early in human embryonic development. During the late two cell zygotic stage, autophagy is actively degrading maternally derived proteins that originated from the oocyte but are now exhausted (Nakashima et al., 2017). The low oxygen tension of the endometrial cavity forces the blastocyst to induce autophagy to achieve extravillous trobphoblast invasion (Genbacev et al., 1997). Subsequent LC3 activity in early pregnancy is also diffuse and ubiquitous (Avagliano et al., 2016), as characterized by the extensive neural tube defects observed when the Ambra1 protein in the PI3K-III complex is absent (Fimia et al., 2007), as well as by strong LC3 signaling in the structures formed by neural crest cells (Cann et al., 2008). Autophagy is also highly active and necessary for neuronal differentiation (Zhao et al., 2010; Avagliano et al., 2016). Experiments have also shown that Atg5 (Mizushima et al., 2001; Kuma et al., 2004; Klionsky et al., 2016) or Atg7 (Komatsu et al., 2005) deletion is characterized by early neonatal death. At its most basic level, autophagy is basally present as an ongoing constituitive process in every cell, though new evidence suggests that different cell types regulate autophagy distinctly (Nakashima et al., 2017).

## Detecting altered fetal brain autophagy activity

It is essential to demonstrate and measure physiologic autophagy first before discussing pathologic autophagy. In commenting on the problem of measuring autophagy activity, Mizushima et al. declared there is “no gold standard” (Mizushima et al., 2010; Klionsky et al., 2016). This is likely due to the myriad of techniques available, including immunohistochemistry, immunoblotting (Jiang and Mizushima, 2015), fluorescence microscopy, electron microscopy, radiolabeling, flow cytometry, and fluorescent probes (Cui et al., 2017; Yoshii and Mizushima, 2017). The autophagy factors most commonly used to measure bilaminar membrane formation and elongation include ULK1, WIPI1/2 [tryptophan (W), aspartic acid (D) repeat domain phosphoinositide-interacting protein], Atg5, and LC3. However, timing when to perform these techniques requires careful planning as autophagic flux may obscure overall trends in autophagy. Measurements of autophagy activity should quantitatively capture its presence or absence. Ideally, measurements should also be recorded over time, so as to reveal trends in the rate of phagosome formation and autophagolysosome degradation. Such intermittent measurement of autophagy activity can pose a challenge. Direct visualization of autophagy vesicles requires electron or immunofluorescence microscopy. Either method requires a cumbersome tissue preparation and image acquisition process that can make the task of creating multiple snapshots to trend autophagosome formation and fusion onerous. Consequently, it is desirable to have insight into the opportune time to perform a measurement so as to capture autophagy activity when it is occurring. For example, Fineschi suggested a model of hypoxia that first uses chaperone proteins such as heat shock proteins (HSPs) and oxygen-regulated protein (ORP150) to detect the onset of the inflammatory response to hypoxia (Fineschi et al., 2017).

The single most common measured autophagy marker is LC3. Avagliano et al. assessed autophagy activity distribution and intensity during development of neural tissue in mouse embryos and human fetuses. Their work revealed similar spatiotemporal autophagy trends using immunofluorescence to detect LC3 expression. One hazard of LC3 measurement lies in that LC3 can be elevated in the context of authophagosome degradation inhibition as well as in ectopy. Autophagy activity can also be in a state of flux. Accordingly, one must measure the change in LC3-II over time (Yoshii and Mizushima, 2017). Co-measurement of LC3-II with degradation of p62 can be used as the latter is directly attached to LC3 and degrades with autophagy (Bjørkøy et al., 2005; Mizushima and Hara, 2006; Pankiv et al., 2007), though p62 should also be used with caution in the setting of starvation (> 2 h) (Jiang and Mizushima, 2015). Alternatively, measuring LC3-II in the presence and absence of autophagy inhibitors such as bafilomycin can be used to increase the accuracy of LC3-II flux measurement (Yoshii and Mizushima, 2017). Radiolabeling amino acids inside cells and then incubating the cells for a time that is long enough to allow proteins with short half-lives to turn over but short enough so as to prevent the reincorporation of freed amino acids into new proteins again facilitates measurement of autphagy flux (Yoshii and Mizushima, 2017).

In another approach to measuring autophagy activity, a tandem fluorescent protein quenching assay joins together RFP (red fluorescent protein), GFP (green fluorescence protein), and LC3 into a single RFP-GFP-LC3B tag (Thermo Fisher Scientific: Waltham, MA). The fluorescence of the tag can be exploited to expose acidic compartments, such as those found in lysosomes. While the green GFP LC3-II and red RFP fluorescence tags together shine yellow and are both present in the neutral pH of the autophagosome, the green GFP LC3-II signal is quenched in the acidic environment of the lysosome, leaving the pH-stable mRFP-LC3 signal to fluoresce red alone (Kimura et al., 2007).

Shvets et al. showed how flow cytometry could be used to detect the levels of fluorescence proteins. The decrease in GFP-LC3 fluorescence reflects ongoing autophagy activity (Shvets et al., 2008).

Each autophagic assay has its own limitations. Ultrastructural analysis (TEM) is insufficient to deal with the biological variability and heterogeneity of an organ/tissue, which requires specialized expertise. Fluorescent microscope or flow cytometry has the potential experimental pitfalls, such as potential for subjectivity, uniform, and criteria for quantitation. Immunoblotting could be less sensitive and informative when analyzing tissue- or stage-specific variations. As autophagy involves dynamic and complicated processes, it is very important to carry out proper assays that deal with the nature of autophagy. All tests that are listed in the Table 1 as well.

**Table 1:** Environmental exposure and autophagy in fetal brain injury.

**Effect**	**Environmental exposure**	**Species**	**Cell Type**	**Autophagy marker**	**Postnatal phenotype**	**Country**	**Author**	**Year**	**PMID**
Inducer	Zika virus	Human	Human fNSC	↓ Akt-mTOR	-Microcephaly -IUGR -Ventriculomegaly -Intracranial calcifications -Spasticity -Seizures -Visual and hearing problems	China	Liang et al.	2016	27524440
Inducer	Zika virus	Mouse	NPCs, neurons, neurospheres and organoids	↑BCL2 ↓Atg12		Brazil	Cugola et al.	2016	27279226
Inducer	Zika virus	Human	Neural stem cells	↓ Akt-mTOR		USA	Chiramel and Best	2017	28899653
Inducer	Rapamycin	Mouse	Mature neurons and glial cells (brain aggregates - bragg)	↓ Akt-mTOR	-Decreased birth weight (RATS) -Decreased fetal viability -Motor impairment	USA	Bajsarowicz et al.	2012	22507918
Inducer	Rapamycin	Mouse	Brain cortical microvessels			France	Girault et al.	2017	28182007
Inducer	Rapamycin	Human	Neurons and astrocytes			USA	Mehla and Chauhan	2015	26198926
Inducer	Rapamycin	Human	Neurons			Italy	Balduini et al.	2012	22385271
Inducer	Hypoxia	*In-vitro*	Neuron	↑LC3 II, ↑ BECN1, ↑Atg7	-IUGR -Microcephaly -Contractures -Arthrogriposis -Hypotonia, lethargy -Feeding difficulties -Apnea -Seizures -Motor deficits	Switzerland	Ginet et al.	2014	24674959
Inducer	Hypoxia	Human	Review article of several cell types	↑LC3-II, ↑Beclin-1, ↑PI3KC3, ↑ATG12-↑ATG-5, ↑p-ULK1		UK	Rocha-Ferreira and Hristova	2016	27047695
Inducer	Hypoxia	Pig	Neurons			China	Cui et al.	2017	28703794
Inducer	PBDE-209	Human	Hippocampus neuron	↑LC3 II ↑Beclin 1	-Lower neurodevelopmental scores	China	Sun et al.	2017	28189061
Inducer	Vitamin D	Rats	Trophoblast cells	↑Beclin 1	-Bone defects -Spontaneous abortion (rats)	China	Tian et al.	2016	26562100
Inducer	Folate Deficiency	Zebrafish	Neural crest cell	↑LC3	N/A	Taiiwan	Kao et al.	2014	25131448
Inducer	Paraquat	Human	Neural progenitor cells	↑Atg5, ↑Atg8, ↑Atg7, ↑Atg12, ↑Beclin-1, ↓mTOR	-Reduced litter size and neurobehavioral and cognitive impairment(mice) -Maternal and fetal death - Chronic lung disease	China	Zhao et al.	2016	27220436
Inducer	MDMA	Mouse	Neuroblastoma cells	↑Atg5	-Hyperthermia -Psycho-Motor deficits -Clubfoot -Congenital heart disease	Korea	Chae et al.	2009	19466606
Inducer	MDMA	Mouse	Neurons	↑LC3		Australia	Mercer et al.	2017	28122248
Inducer	Fluoxetine	Rats	Neurons, microglia and astrocyte	↑Beclin-1	-Respiratory distress -Cyanosis, apnea -Seizures -Irritability -Tremor -Feeding difficulties and vomiting -Hyper/hypotonia -Hyperreflexia	China	Li et al.	2017	28903766
Inducer	Dopamine	Rat	Embryonic cortical neurons	↑LC3 II	-Cardiac malformations (chicks) -Embryotoxicity (rats)	Taiwan	Hung et al.	2017	28427888
Inducer	Chitosan	Zebrafish	Fibroblasts and neural stem cells (nscs)	↑Atg 5, ↑Atg7, ↑LC3 II	Unable to locate data	Taiwan	Tseng et al.	2016	26815305
Inducer	Endothelial Reticulum Stress	Human	CNS cells	Atg4, Atg9, Atg10, Beclin1, LC3, PI3KC3	N/A	USA	Yang and Luo	2015	26473940
Inducer	Reactive Oxygen Species	Mouse	Neuronal stem cells	↑LC3-I, ↑LC3-II, ↑Atg9	N/A	Portgual	Fonseca et al.	2013	23729317
Inducer	Starvation	Rats	Hippocampal neurons	↑Atg 3, ↑Atg7, ↑Beclin 1, ↑p-AKT, ↑p-mTOR	-Decreased neonatal weight	China	Feng et al.	2012	21905985
Inducer	Hyperglycemia	Chick	Cranial neural crest cells	↑LC3-I → II, ↑fluorescence of pGFP-LC3 ↑Atg9	-Cardiovascular, neurologic, gastrointestinal, genitourinary, skeletal anomalies	China	Wang et al.	2015	26671447
inducer	Glucose	Mouse	Oligodendrocytes and corpus callosum	↑LC3 II		USA	Lei et al.	2017	29017418
Inducer	Ethanol (acute)	Mouse	SH-SY5Y neuroblastoma cells	↑LC3 II ↑BECN1	-Fetal alcohol syndrome	USA	Chen et al.	2012	22874567
Inducer	Ethanol (acute)	review	neurons	mTOR, AMPK, Bcl2		USA	Luo	2014	25484085
Inducer	Ethanol (acute)	Human	CNS cells	↑Atg4, ↑Atg9, ↑Atg10, ↑beclin1, ↑LC3 perk, ↑ pik3c3		USA	Yang and Luo	2015	26473940
Inducer	Ethanol (acute)	Human	Neurons	↑Autophagosomes in TEM		Japan	Eto et al.	1992	1520402
↑apoptosis ↓autophagy	Ethanol (chronic)	Mouse	Neurons	↑Atg4, ↑Atg9, ↑Atg10, ↑ beclin1, ↑PIK3C3, ↑ LC3		USA	Alimov et al.	2013	23979425
↑apoptosis ↓autophagy	Ethanol (chronic)	Mouse	Brain cortical microvessels	↑ GFP-LC3		France	Girault et al.	2017	28182007
↑apoptosis ↓autophagy	Ethanol (chronic)	Mouse	Cortical neuroepithelial cells	↑vacuole formation		USA	Prock and Miranda	2007	17374049
Inhibiter	Sitagliptin	Mouse	Neurons	↓LC3B-II	Unable to locate data	Egypt	Nader et al.	2018	29032011
Inhibiter	Glucose	Mouse	Neuroepithelium	↓LC3-GFP green puncta		USA	Wang et al.	2017	28474670
Inhibiter	phencyclidine	Rats	Cortex and hippocampus	↓Beclin 1	-Dysmorphic fascies -Jitteriness, hypertonia, vomiting and diarrhea	Serbia	Jevtić et al.	2016	26655035
Inhibiter	Wortmannin	*In-vitro*	Hippocampal neurons	↓GFP-LC3	Unable to locate data	China	Chen et al.	2013	24094936
Inhibiter	3-MA (3- Methyladenine)	*In-vitro*	Hippocampal neurons			China	Chen et al.	2013	24094936
Inhibiter	chloroquine	*In-vitro*	Hippocampal neurons		-low birth weight	China	Chen et al.	2013	24094936
Inhibiter	Bafilomycin	Human	Neuroblastoma cells	↑mTOR	Unable to locate data	USA	Chen et al.	2012	22874567
Inhibiter	Bafilomycin	Human	Glioma stem/progenitor cells (gspcs) and neural stem/progenitor cells (nspcs)	↑mTOR		China	Zhao et al.	2010	20004652
Inhibiter	Spermidine	Rats	Neurons	↓Beclin 1 ↓LC3-II		China	Zhang et al.	2017	28112032
Inhibiter	Green Tea Theanine	*In vitro* (murine)	Neural progenitor cells	↑mTOR	Unable to locate data	Japan	Takarada et al.	2015	28955810
Inhibiter	Acai fruit pulp extracts	Rat	Hippocampal neurons	↑mTOR	Unable to locate data	USA	Poulose et al.	2014	24985004

*Current progress of autophagy associated with fetal brain injury were described in Table, including effects, environmental exposure, species, cell type, methods and conclusions. References for phenotypes column: Reprotox database*.

## Discussion of environmental exposures

Multiple different environmental exposures alter autophagy activity in the fetal brain (Table 1). Broadly, many of them can be characterized as infectious, hypoxic, and toxic. While the mechanism detailing the pathway from affect to phenotype is urgently needed, at least some evidence shows that autophagy influences these phenotypic presentations.

Multiple infectious agents alter autophagy as part of their pathogenic exertion. The Zika virus (ZIKV) may cross the placental barrier via a special type of autophagy called secretory autophagy (Zhang Z. W. et al., 2016). Once crossed, the virus hones in on tropic factors (Miner and Diamond, 2017) to infect human fetal neural stem cells (fNSCs). Once inside an fNSC, ZIKV makes NS4a and NS4b proteins that decrease mTOR and induce autophagy (Liang et al., 2016) in a way that impairs neurogenesis (Chiramel and Best, 2017). ZIKV also causes microcephaly via apoptosis and autophagy-driven cell death of cortical progenitor cells (Cugola et al., 2016). Research has shown that treatment with the autophagy inhibitor hydroxychloroquine reduces the incidence of ZIKV vertical transmission in pregnancy (Cao et al., 2017).

Similar to Zika, HIV also exerts its some of its effects via autophagy. Once HIV has infected an astrocyte, it produces a Nef protein that is associated with the HIV-Associated Neurocognitive Disorders (HAND) phenotype. It has been shown that infecting fetal astrocytes with an adenovirus based vector of the Nef protein leads to accumulation of autophagosomes by way of blocking their fusion with lysosomes. As autophagy has an essential role in innate immunity, the manufacture of Nef by HIV safeguards its presence inside the cell and allows it to avoid destruction by the lysosome's enzymatic and acidic environment (Saribas et al., 2015).

While hypoxia and infection may be the result of different underlying etiologies, they share a common disease pathway in autophagy. The exact role of autophagy in neonatal hypoxic-ischemic encephalopathy (HIE) is controversial. The findings in several rodent studies have demonstrated conflicting roles for autophagy, with some data suggesting a cell-protective role (Carloni et al., 2008; Wang et al., 2012) while other studies suggest autophagy led to a cell-death pathway (Koike et al., 2008; Wen et al., 2008; Bidlingmaier et al., 2009; Xing et al., 2012). Moving away from the rodent model, Ginet et al. has shown in late pre-term and term neonates that HIE increased the number of autophagosomes and lysosomes by one order of magnitude in the asphyxia-sensitive ventrolateral thalamic region of the brain. They demonstrated comparable findings in both rodent and human neonates who died after acute perinatal HIE. Their collected biomarker data also demonstrated increased autophagy activity with significant increases in LC3-II activity and simultaneous decreases in p62 (Ginet et al., 2014).

In addition to the autophagy mediated hazards posed by infection and hypoxia, toxins also potently influence autophagy. For example, exposure to ethanol during the ethanol sensitive period of pregnancy leads to an increase in apoptosis and a decrease in autophagy. Earlier research in humans suggested that the ethanol sensitive period of pregnancy was as late as 20 weeks of gestation. More recent data from a rodent model shows less autophagy activity and increased apoptotic markers when mice are exposed to ethanol on post natal day 4, a time period which approximately correlates to the third trimester in humans. Conversely, later in pregnancy, when the fetal brain stress response system has matured, ethanol exposure leads to an increase in multiple autophagy markers that are accompanied by significantly fewer apoptotic markers, signaling the onset of an ethanol resistant period in pregnancy (Alimov et al., 2013).

In addition to gestational timing, ethanol exposure has a second temporal affect on autophagy in the fetal brain. Acute ethanol exposure induces autophagy activity to protect the developing brain. In contrast, chronic ethanol exposure in adult progeny activates mTOR, thereby inhibiting the autophagy pathway in the brain (24556681). Consequently, chronic ethanol exposure may impair protective autophagy function when fetal neurons are faced with increased stress.

A second toxin associated with recreational drug use is 3,4-methylenedioxymethamphetamine (MDMA). It is also used to treat post traumatic stress disorder (PTSD) (Amoroso and Workman, 2016). MDMA has been shown to upregulate autophagy in the fetal brain by increasing Atg5 and LC3 levels (Chae et al., 2009). Consuming MDMA during pregnancy is related to fetal neural and cardiotoxicity as well as impaired motor functioning (Meamar et al., 2010).

Though now less commonly abused, phencyclidine (PCP) acts as a non-competitive antagonist of the glutamatergic N-methyl-d-aspartate (NMDA) receptor. Its administration to pregnant rats alters the behavior of their offspring and causes neurodegenerative effects similar to those seen in schizophrenia (SCH) (Radonjić et al., 2008). Autophagy has also been shown to play a key role in the disease mechanism of SCH (Merenlender-Wagner et al., 2015). A recent study shows the presence of autophagy in a PCP model of SCH in rodents. After PCP administration, autophagy downregulation was seen by way of reduced Beclin1 expression in the neocortex and in the hippocampus (Jevtić et al., 2016).

While some maternal exposures are part of the spectrum of substance abuse, others are so ubiquitous that they are difficult to avoid. For example, paraquat is a commonly used herbicide. Paraquat exposure to a human progenitor cell line is associated with an increase in autophagy (Zhao et al., 2016). In the adult phenotype, paraquat has been linked to Parkinson's disease in farm workers through an increased production of reactive oxygen species exerting their toxicity on neurons (Tanner et al., 2011).

A final category of substances that alter autophagy brain activity in cells is very small metal particles. Metals, due to their nature, cannot be degraded by a lysosome's enzymatic complement. Consequently, the entry of micronized metals into a cell may induce autophagy though the digestive process cannot be completed. Instead, the particles accumulate in autophagosomes. Researchers have raised concerns about the impact the fetal and neonatal brain may suffer from altered autophagy activity from exposure to silver (Guo et al., 2017) and titanium dioxide nanoparticles (Song et al., 2016), as well as even smaller Cadmium Selenide / Zinc Sulfide (CdSe/ZnS) quantum dots (Chen et al., 2013).

The previous discussions on various exposures are not exhaustive and we cannot explain the mechanism for all the exposures and phenotypes listed in Table 1. However, we can reasonably speculate that autophagy plays at least some role in certain phenotypic presentations. For example, progeny with features of decreased physical size, such as microcephaly, low birth weight, or IUGR may be attributable to elevated autophagy levels. The psycho-sensorimotor deficits seen with certain exposures may also be attributable to dysregulation of autophagy activity in neurons.

## Future investigations

All markers of autophagy activity cited in Table 1 were recovered post-mortem or *in-vitro*. Future research is urgently needed to employ these markers for *in utero* identification of potential disease processes associated with environmental exposures in pregnancy.

While many environmental exposures that influence autophagy activity have been identified, many remain to be discovered. The discovery of additional safe and economical *in vivo* modulators of autophagy would offer additional tools to reverse common pathogenic affecters of autophagy.

Three promising treatments for altered autophagy levels include glucose, modest hypoxia, and hydroxychloroquine.

Our recent discovery of glucose infusion for pathogenic autophagy activity represents a potentially accessible and cost-effective treatment of perinatal brain injury in the setting of intrauterine inflammation secondary to chorioamnionitis or preterm birth (Lei et al., 2017).

A potential treatment for chronic ethanol exposure could lie in the development of safe autophagy inducers. This has already been demonstrated in a limited way by a study which showed that a modest hypoxic preconditioning induced protective autophagy in human neuronal stem cell cultures affected by long term exposure to alcohol (Luo, 2014).

While the peak of the recent Zika outbreak in the Americas has passed, it is imperative that further research is performed about its management. Attempting to affect its clinical course with the use of autophagy modulating molecules such as hydroxychloroquine or possible acai derivatives offers new areas for research.

Finally, further studies are needed in order to elucidate the causal connections between altered embryonic and fetal autophagy activity and psychiatric disorders.

## Author contributions

IB: Designed review and selected articles for the synopsis, guided manuscript structure, and supervised writing: PC: Reviewed literature and participated in the writing of the manuscript; RV: Reviewed literature and participated in the writing of the manuscript; JL: Helped in article selection for the review, reviewed literature, and participated in the writing of the manuscript.

### Conflict of interest statement

The authors declare that the research was conducted in the absence of any commercial or financial relationships that could be construed as a potential conflict of interest.
